# Investigation and Optimization of Effects of 3D Printer Process Parameters on Performance Parameters

**DOI:** 10.3390/ma16093392

**Published:** 2023-04-26

**Authors:** Ray Tahir Mushtaq, Asif Iqbal, Yanen Wang, Mudassar Rehman, Mohd Iskandar Petra

**Affiliations:** 1 Bio-Additive Manufacturing University-Enterprise Joint Research Center of Shaanxi Province, Department of Industry Engineering, Northwestern Polytechnical University, Xi’an 710072, China; tahirmushtaqray@mail.nwpu.edu.cn (R.T.M.); mudassar@mail.nwpu.edu.cn (M.R.); 2 Faculty of Integrated Technologies, Universiti Brunei Darussalam, Jalan Tungku Link, Gadong BE 1410, Brunei; iskandar.petra@ubd.edu.bn

**Keywords:** additive manufacturing, fused filament fabrication, parametric optimization, mechanical properties, energy efficiency

## Abstract

Professionals in industries are making progress in creating predictive techniques for evaluating critical characteristics and reactions of engineered materials. The objective of this investigation is to determine the optimal settings for a 3D printer made of acrylonitrile butadiene styrene (ABS) in terms of its conflicting responses (flexural strength (FS), tensile strength (TS), average surface roughness (Ra), print time (T), and energy consumption (E)). Layer thickness (LT), printing speed (PS), and infill density (ID) are all quantifiable characteristics that were chosen. For the experimental methods of the prediction models, twenty samples were created using a full central composite design (CCD). The models were verified by proving that the experimental results were consistent with the predictions using validation trial tests, and the significance of the performance parameters was confirmed using analysis of variance (ANOVA). The most crucial element in obtaining the desired Ra and T was LT, whereas ID was the most crucial in attaining the desired mechanical characteristics. Numerical multi-objective optimization was used to achieve the following parameters: LT = 0.27 mm, ID = 84 percent, and PS = 51.1 mm/s; FS = 58.01 MPa; TS = 35.8 MPa; lowest Ra = 8.01 m; lowest T = 58 min; and E = 0.21 kwh. Manufacturers and practitioners may profit from using the produced numerically optimized model to forecast the necessary surface quality for different aspects before undertaking trials.

## 1. Introduction

Additive manufacturing (AM), also known as 3D printing, signifies constructing a physical object by adding material layer upon layer based on a digital model [1,2]. This is accomplished using desktop design (CAD) software. The AM methods are based on the concepts of starting with a modeled contains simple, slicing the created files into two-dimensional sectors, and transporting them to the Am platform. The implementation of AM has progressed beyond the prototyping stage and is now a highly adaptable manufacturing process. AM relies entirely on rapid prototyping to construct intricate and substantial structures using 3D CAD software [3]. Sheet lamination, deposition, selective laser [4,5,6,7,8], fused deposition modeling, and stereolithography are only some of the additive manufacturing (AM) techniques that may be utilized to produce the material element layer by layer [9,10,11]. Parts built by Laser-Powder Bed Fusion offered significant advantages for aerospace applications; the validation of their mechanical properties remains an important research area to ensure their safety and effectiveness in critical applications [12].

**Figure 1:**
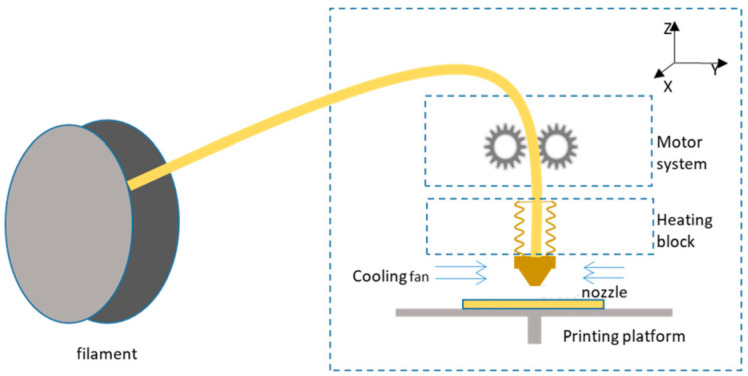
Schematic of FFF-based 3D printer (figure reprinted from [16] under the license CC-BY 4.0).

In 1988, under the name fused deposition modeling (FDM), Crump [13] registered the fused filament fabrication (FFF) process of 3D printing. This laid the groundwork for establishing the late-20th-century Stratasys business in 1989. FFF is unique in its ability to create complex shapes. Electrical motors power the feeding of a filament into a molten pool in this additive manufacturing technology. Stepper motors then drive the printer head over a platform. Ink is deposited in the “X” and “Y” planes [14] after being liquefied and pushed through the printer nozzle. The printer platform is lowered into the “Z” track as each successive cross-section is carefully put. As a result, the 3D-printed design is built up in layers [15]. To complete the sample, just repeat the procedure [10]. The FFF printer’s operational scheme is shown in Figure 1 [16].

When quick manufacturing of devices was needed as part of the emergency reaction to COVID-19, three-dimensional printing (3DP) was employed as a transportable factory [17]. Among the many fields that can benefit from technology, we find the medical/dental/aviation/refrigeration/automotive sectors [18,19]. Dental models using AM techniques such as FFF and Poljet are precise and accurate [20,21]. Individualized prosthetic devices, improved manufacturing processes, fiber-reinforced structures for automobiles and airplanes, conductive structures and implants, biological and construction tools, physiochemical health products, the fine jewelry and gemstone industries, soot particle filters and lightweight heating devices, and investment artifacts are all examples of uses for FFF that have been studied by the authors [22,23,24,25,26]. FFF 3DP has several potential uses, such as increasing the tensile strength of materials [27,28], making automobile parts [29], making research prototypes [30,31,32], analyzing microstructures [33,34], and combating the COVID-19 virus [35,36,37].

Thermoplastic polymer filaments made with the FFF feedstock method are the most widely used printing materials. There are many challenges that the FFF method is always up against. Because it has limitations in terms of mechanical strength, i.e., flexural strength (FS) and tensile strength (TS), print time (T), average surface roughness (Ra), and energy consumption (E) [38]. To improve the FDM process, a lot of work has gone into developing new FFF feedstock materials, honing the process parameters, and studying various parts’ mechanical, thermal, and rheological properties. Only thermoplastics may be used as input materials when using the FFF’s heating and extruded filament through the extruded nozzle. Polypropylene, polylactic acid, poly-phenyl-sulfone, nylon, polyethylene, and polycarbonate are just some of the polymers composite that FDM can print [39]. This research aims to determine what influences the Fabrication technique parameters the most so that better mechanical characteristics, Ra, T, and E, may be achieved in 3D-printed objects. Acrylonitrile butadiene styrene (ABS) has been used in the medical field [40], in tissue engineering [41], in the airline industry [42], and in the car industry [25]. The automotive sector requires more robust 3D-printed products, molds, and fixtures. However, problems such as subpar mechanical qualities, subpar surface finish, and a lengthy production time still need to be fixed. It is envisaged that the findings of this investigation would be applicable in ensuring that 3D-printed ABS components achieve the required tensile strength in a reasonable amount of time. Additive manufacturing technologies must reduce lead and production times to compete with traditional manufacturing processes. Since improved surface and mechanical properties are required to manufacture functional components, the construction time must also be decreased. Unchecked “failures”, such as blocked nozzles, might significantly lengthen the construction schedule. The process parameters also affect the time spent building FFF parts; selecting the right set of values might help. Thus, there is a lack of investigation into how energy usage affects energy efficiency, carbon pollution, and the price of producing an item.

The relationships between print quality (PS), layer thickness (LT), and infill density (ID) have been studied [43,44]. LT is one of the most studied properties of FFF 3D-printed items [45]. The overall mechanical characteristics of FFF parts in 100% ID have been demonstrated to be improved by the LT mid-level [46]. Mechanical response measurements and other 3D printing features are less visible. The LT is impacted by changes in heat transfer between the freshly deposited material and older, previously placed material [30]. At the highest PS, surface quality deteriorates and mechanical characteristics decrease, but with the interactions of high ID, PS acquires substantial stiffness [47]. The final product’s porosity is established by the ID [48]. The FS and TS of the components improves with increasing ID [49]. It is projected that the 100% ID will have a higher mechanical response. Existing research [48] indicates that PS, LT, and other control parameters affect the porosity and dynamic response in 100% ID.

Although there are very important optimization techniques, such as numerical analysis using finite elemental analysis for simulations [50], RSM multi-objective optimization has become one of the most used optimization methods due to the need for physical optimization and because it is a useful instrument for adjusting AM procedure settings. The RSM method is used for optimization in several contexts, most notably in computer numeric control, with various techniques, such as [51] “laser processing” [52], “electric discharge machining” [53], etc. Griffiths et al. mentioned that a small experimental error rate makes the RSM a great optimization tool. [54]. The RSM method may be better for optimizing multi-response 3D printers, because it can be modeled with higher fitting and multi-objectivity [55]. The FFF processing settings were examined and adjusted by Selvam ani et al. [56], who used RSM analysis. The highest mechanical property values observed in experiments were 0.333 GPa for elastic modulus, 7.758 MPa for ultimate tensile strength, and 4.539 MPa for yield strength.

Therefore, to fill the gaps, this research examines the in-depth effect, ID, and PS (by taking minimum to maximum levels) on the FS, TS, Ra, T, and E performance parameters of the FFF-3DP ABS subcomponents. This investigation thoroughly explores several conflicting performance parameters important for industrial applications, including mechanical characteristics, power efficiency, and surface quality enhancement. According to its proponents, ABS K5 filament is superior to its commodity counterpart in printability, ease of use, and lack of shrinkage, wrapping, warpage, mechanical characteristics, and printability. To complete the research, the authors will follow these procedures:

- Analyze the regression analysis and the magnitude of the parametric influence on performance parameters using ANOVA;
- Investigate how changing LT, ID, and PS values affect ABS’s FS, TS, Ra, T, and E;
- We can achieve optimal performance by applying RSM’s multi-objective numerical optimization technique to the FFF 3DP’s parameters;
- Conduct trials and analyze the results using an SEM to verify the optimum sample preparation.

## 2. Materials and Methods

### 2.1. Materials

The investigations were conducted using an industrial, professional 3D printer called “CR-5” manufactured by Creality, Shenzhen, China, whose properties are shown in Table 1, and industrial ABS (ABSK5) polymer with 1.75 mm diameter filament procured from the KEXCELLED firm, Suzhou, China. Table 2 contains ABS material specifications.

### 2.2. Response Surface Methodology

The methodology and experimental design (DOE) are described in this section. The studies were designed and conducted using a full RSM-central composite design (RSM-CCD). Several printing process parameters substantially influence the diverse performance parameters that are researched, mainly with respect to FFF 3D printing. After thoroughly reviewing the relevant literature, LT, PS, and ID were determined to be the most important quantitative input factors. Material, mechanical properties, the design process, and sample geometries affect the selected range. Therefore, the printing parameters were determined based on previous academic data and early tests [46,48].

The experimental parameters and factors were determined using the statistical software, Design-Expert version 13, which is widely recognized for its advanced features in experimental design and analysis. Quantitative parameter settings (LT, PS, and ID), for a thorough analysis, comprise five parametric levels each. To avoid decimal values in the maximum and lowest parameters, L20 DOE was designed with a full CCD and alpha 1.5. After sending the STL file to the slicer, establishing the parameters, and slicing the model, it was produced on a 3D printer. After calculating the performance parameters, the values were placed into a CCD table created by a design expert. Table 3 shows the detailed parametric experimental design for the ABS polymer.

**Figure 2:**
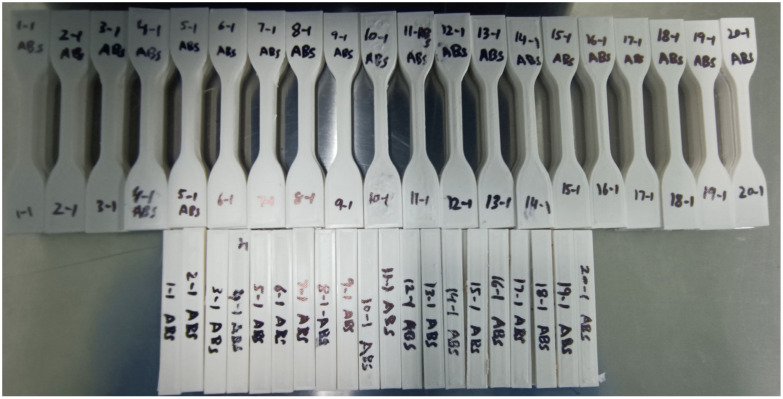
FFF fabricated experimental samples for the ABS polymer.

Figure 2 demonstrates the manufactured ABS sample models for ISO 527 and ISO178, respectively. The authors printed three samples for each test, conducted FS and TS tests on each of the three ISO 527 and ISO 178 samples, calculated the mean, and measured three Ra values before calculating the mean.

The authors determined the minimum PS to be 47 mm/s and the maximum PS to be 75 mm/s. CCD measured a lower value of 40 mm/s and a higher parameter of 82 mm/s. At a PS of less than 40 mm/s, obtaining three samples needed more than 10 h, which was inefficient. Due to insufficient cooling time, a high PS results in poor layer adhesion. When the PS is reduced to extremely low levels, part distortion occurs. Thus, the minimum and maximum speeds would be 47 mm/s and 75 mm/s, respectively. The line thickness was often measured between 0.14 mm and 0.3 mm, but CCD measured one parameter as 0.1 mm and another as 0.34 mm. As the nozzle was 0.4 mm, LT’s maximum 3D printing range was limited to 0.34 mm, while ABS printing was impossible below 0.1 mm. Thus, the authors chose 0.1 as the minimum ABS value. To thoroughly comprehend the ID, the authors used a lower limit of 20 percent and an upper bound of 84 percent. In comparison, CCD used a lower parameter of 4 percent and a higher parameter of 100 percent.

#### Measurement Procedure

**Figure 3:**
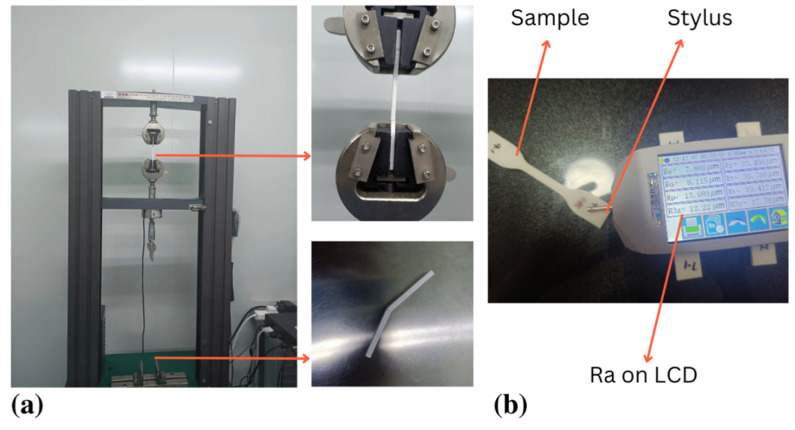
FFF experimentation. (**a**) TS and FS testing, (**b**) Ra testing.

The FS and TS tests were complemented by a GTM 2500 Equipment with a 5-KN weight cell, as shown in Figure 3a,b. Following ISO 527: 1997, TS tests were accompanied on plastics at a cross-head speed of 5 mm/min [57]. ISO 178:2006 (plastics: calculating FS characteristics) was used to determine FS. The prescribed standards for TS specimen dimensions in testing demand precise attention to detail, requiring an overall length of 115 mm, a gauge length of 25 mm, a width of 19 mm, a thickness of 4 mm, and a length of the narrow section of 33 mm. Meanwhile, the standards for the TS test were a width of 10 mm, a length of 80 mm, and a thickness of 4 mm. The specimen is held in situ by two supports 64 mm apart and subjected to a central weight until it fractures. The 5-mm radius spherical equipment is the loading head. The tests employed 25 °C and 2 mm/min cross-head speed [58,59].

As illustrated in Figure 3b The Ra value of the FFF-fabricated component was evaluated using a ‘JITAI KEYI’ Ra tester (JD520 model). In Equation (1), the Ra value can be defined as the arithmetic average of all actual values of variations in the surface profile measured along the whole distance from the centerline. The ISO 16610-211 standard was employed for analysis, with sample lengths (Ls) of 4.8 mm and cut-off wavelengths of 0.8 mm [60].

(1) $$Ra=\frac{1}{La}\int_{0}^{La}\left|Z\left(x\right)\right|dx$$

where *La* is the sampling length and *Z*(*x*) organizes the profile curve.

## 3. Results and Discussion

### 3.1. ANOVA for Performance Parameters

A design expert analyzed the present investigation’s regression model and the ANOVA approach (version 13). Following the computation of the regression analysis, the final regressions regarding the actual values for FS, TS, Ra, T, and E were created as follows:

FS = 29.512 + 342.237 × LT − 0.045 × ID + 1.276 × PS + 0.550 × LT × ID − 1.828 × LT × PS + 0.004 × ID × PS − 521.833 × LT^2^ − 0.001 × ID^2^ − 0.009 × PS^2^ (2)

TS = 40.031 − 23.622 × LT + 0.004 × ID − 0.383 × PS − 0.437 × LT × ID + 0.648 × LT × PS + 0.001 × ID × PS + 48.307 × LT^2^ + 0.002 × ID^2^ + 0.002 × PS^2^ (3)

Ra = −6.340 + 41.196 × LT − 0.038 × ID + 0.205 × PS + 0.075 × LT × ID − 0.126 × LT × PS + 0.000 × ID × PS − 37.243 × LT^2^ − 0.000 × ID^2^ − 0.001 × PS^2^ (4)

T = 234.365 − 817.515 × LT + 0.551 × ID − 2.142 × PS − 1.562 × LT × ID + 2.232 × LT × PS − 0.001 × ID × PS + 1177.510 × LT^2^ + 0.001 × ID^2^ + 0.009 × PS^2^ (5)

E = 0.863 − 3.100 × LT + 0.003 × ID − 0.008 × PS − 0.006 × LT × ID + 0.010 × LT × PS − 0.000 × ID × PS + 4.295 × LT^2^ + 0.000 × ID^2^ + 0.00003 × PS^2^ (6)

ANOVA was used to approximate and assess the regression coefficients to determine which process characteristics significantly influence the dynamic qualities. Table 4, Table 5 and Table 6 summarizes the ANOVA results for all performance parameters. Table 4 shows the mean and standard deviation for the sum of performance parameters, and Table 5 shows the standard deviation at each point. The F-statistics for FS = 46.09, TS = 25.45, Ra = 89.64, T = 196.35, and E = 150.26, respectively, and the *p*-values in all performance parameters were less than 0.05 for the second-order regression model as seen in Table 6. The *p*-values for the lack-of-fit were calculated for FS = 0.004, TS = 0.556, and Ra = 0.3174, respectively. This shows that the lack-of-fit is small compared to a residual error. A small lack-of-fit is a good sign because it indicates that the terms left out of the model are insignificant. *p*-values for the lack-of-fit for T and E were not available. This implies that the lack-of-fit is minimal or non-existent for TS, T, and E, but significant for TS and Ra of residual error. The determination coefficient examines the goodness-of-fit of the regression models. It is preferable to have a larger prediction coefficient (close to 1.0). For all parameters, the created relationship had a significant value of R^2^, the adjusted R^2^, and the anticipated R^2^, indicating a good correlation between the experimental and calculated values. A number greater than four is preferred. In this investigation, the appropriate precision for FS and TS is greater than 4, indicating a sufficient signal.

**Figure 4:**
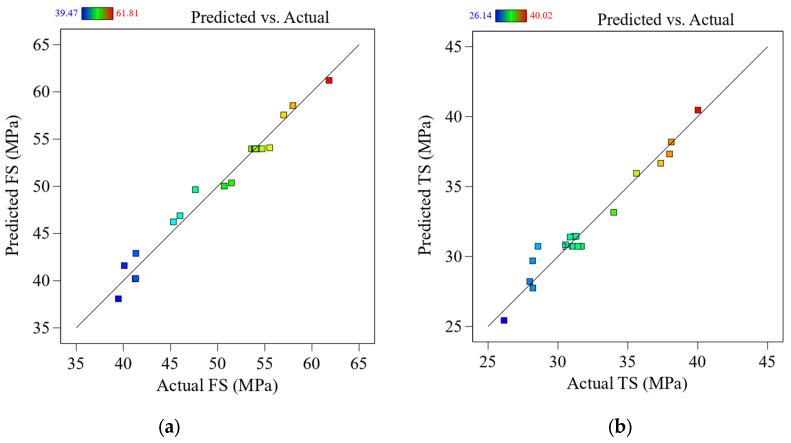

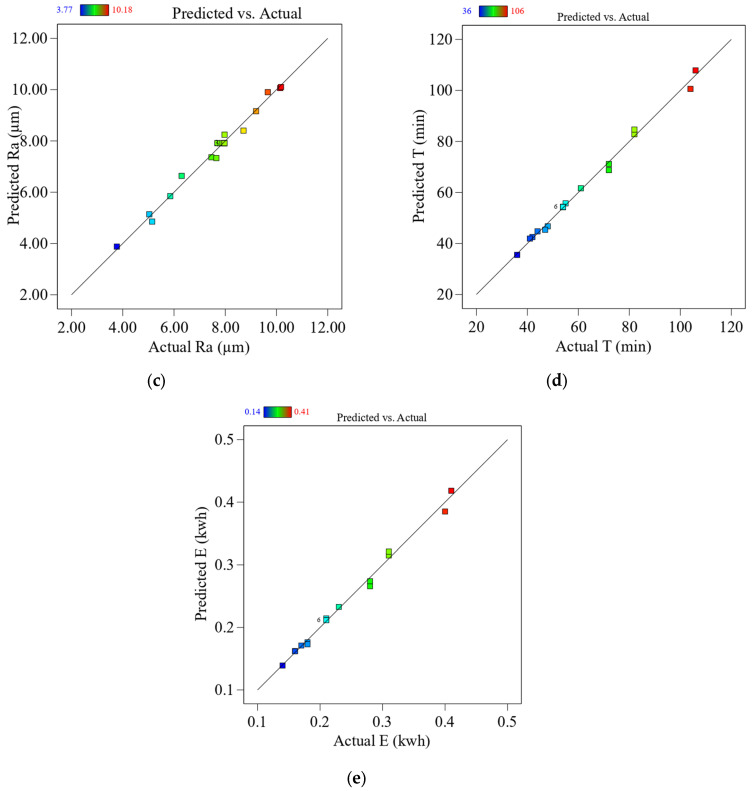
Predicted vs. actual performance parameters for (**a**) FS, (**b**) TS, (**c**) Ra, (**d**) T, and (**e**) E.

Before reaching any stronger conclusions, performing a residual analysis to test the assumptions behind the ANOVA results is critical. This is shown in Figure 4a–e as a comparison between the expected and experimental values for the FS/TS, Ra, T, and E. According to the experimental results, the predicted values are extremely close to the actual ones. To put it another way, this shows that existing models can accurately predict the answers.

### 3.2. Printing Parametric Effects on Mechanical Properties

**Figure 5:**
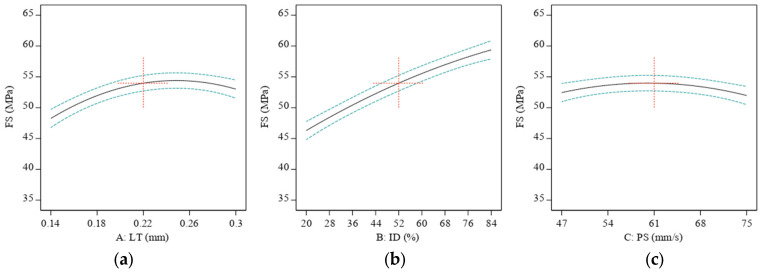

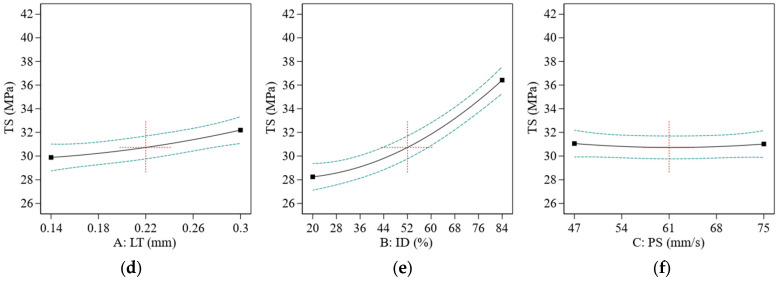
The effect of printing parameters on ABS: (**a**) FS vs. LT, (**b**) ID vs. FS, (**c**) PS vs. FS, (**d**) LT vs. TS, (**e**) ID vs. TS, and (**f**) PS vs. TS. Red represents center point. Black is the average data line and green lines show the min and max of data.

Thermoplastic polymers such as ABS are used in FFF-based 3D printing. The material is heated to a moderate condition before extruding through the nozzle to create the desired three-dimensional shape. The LT utilized in the printing may influence the interfacial bonding or the strength of the link between neighboring layers in the 3D-printed item. Due to the bigger LT’s ability to reduce non-uniform temperature gradients, deformations, and thermal stresses, it may be possible to reduce the required heat cycles [61]. Figure 5 shows this weak interlayer link might cause delamination or part failure for 3D-printed components exposed to severe loads or pressures—thinner inter-layer bonding results in a lesser void volume fraction, eventually giving higher strength to the material. The 3D-printed component’s strength and integrity may be increased, and its resistance to failure under stress can increase. However, the microlayers are fragile, and the thinner layers induce movement at the lowest LT. Thus, 0.25 mm of LT is where it is at for FS. Figure 5d shows that even at the maximum level of LT, the LT was less of a factor in TS because the LT provided less area to the sample, and there were fewer microstructure concerns.

The mechanical qualities of a 3D-printed item may be affected by the ID of the component, which is the total quantity of material utilized to fill the part’s interior. This means that increasing the ID of a 3D-printed item for FS and TS made it stronger and stiffer, as seen in Figure 5b,e. This is because an increase in ID implies an increased amount of material present to balance stresses throughout the item.

A 3D-printed object’s mechanical qualities may change depending on how quickly it was created. After a specific threshold, raising the printing speed yields a weaker and less exact final item, while reducing the PS yields a stronger and more specific object, as shown in Figure 5c for TS. A 3D printer’s output quality suffers when the extruded polymer is not given enough time to cool and harden before adding another layer. The final product may have less strength because of the weaker link between the layers. Furthermore, the extruded polymer may lose accuracy due to the rapid printing pace.

When 3D printing at a low speed, the printer’s nozzle moves slowly, which means that the material being deposited has more time to absorb heat from the nozzle. As a result, the material becomes softer and more pliable, making it easier to bond with the layers below it. This process is known as “bonding strength development at the interface”. However, the nozzle’s slow movement can increase the material’s residual stresses, reducing the material’s strength [49,50].

(7) $$\ln\left(\sigma \right)=\frac{-1}{n}\ln\left(PS\right)+C$$

This equation is derived from the polymer healing theory equation, where *C* is a constant.

Despite this risk, printing at a low speed can be beneficial in certain situations, for example, when the printed material is prone to cracking or has already developed small cracks. In that case, printing at a low PS could help to “heal” these cracks by allowing the material to bond more strongly at the interface. This healing process may not be complete, but it could still improve the overall strength of the object, as shown in Figure 5f for TS. Figure 5f showed the highest TS at the midpoint, and then further increment of PS decreased the value of TS.

The optimum printing speed for a given 3D-printed ABS polymer object will depend on its intended use and the desired mechanical properties. Objects that require high strength and precision, such as mechanical parts or structural components, will benefit from a lower printing speed. Objects that do not require high strength and precision, such as prototypes or decorative items, can be printed at a higher speed without sacrificing performance.

**Figure 6:**
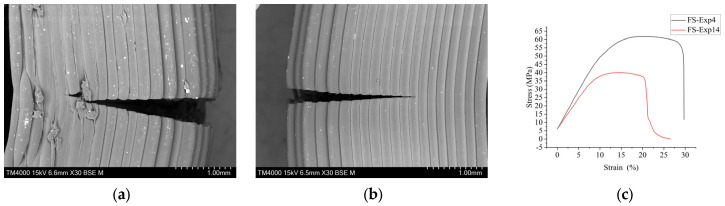

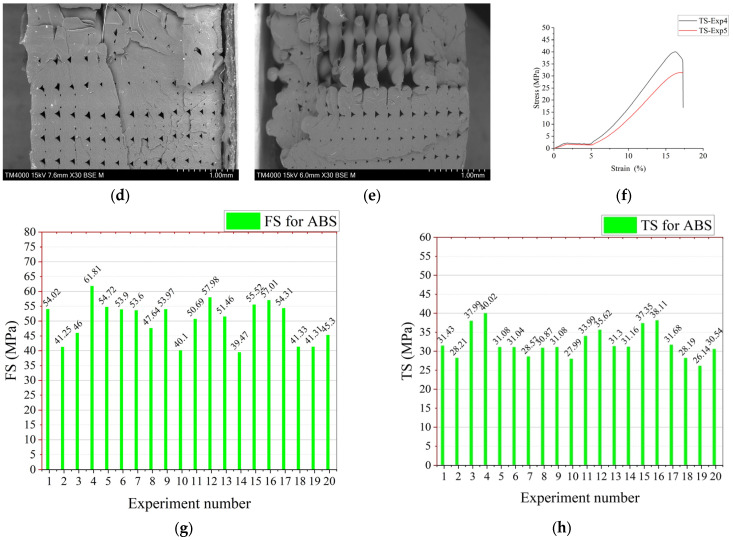
SEM images of TS and FS samples: (**a**) FS-Exp 4 (ID = 100%, PS = 75 mm/s, LT = 0.22 mm), (**b**) FS-Exp number 14 (ID = 20%, LT = 0.3 mm, PS = 75 mm/s), (**c**) Stress strain curve for Exp 4 and 14, (**d**) TS-Exp 4 (ID = 100%, PS = 61 mm/s, LT = 0.22 mm), (**e**) TS-Exp 5 (ID = 52% PS = 61 mm/s, LT = 0.22), (**f**) Stress strain curve for Exp 4 and 5, and (**g**) FS results for ABS (**h**) TS results for ABS.

Figure 6 shows the SEM images taken for TS and FS samples. Figure 6a,b show the SEM samples at the highest and the lowest values of FS, respectively. The highest level of FS was obtained in experiment 4. Figure 6a shows little distortion in layers, bulges, and micro crack due to some humidity in material [59,62]. It shows well-packed layers with 100% ID, which give bigger strength to the sample, and some layers were so packed that they were coming out of the samples, as shown in the micro-SEM image of Figure 6a. Figure 6b shows the FS sample at the lowest FS at experiment number 14 with an FS value of 39.47 (see Figure 6g), at the lowest value of ID as 20% and high level of thickness as 0.3 mm and PS at highest value as 75 mm/s. Figure 6c shows the maximum and minimum FS values of the stress–strain curve.

Figure 6d shows the TS sample at the highest level of TS in experiment 4. It shows well-packed layers with 100% ID, giving the sample bigger strength. TS showed the lowest value at experiment number 19 with a TS value of 26.14 MPa at the lowest value of ID as 20% and of thickness as 0.14 mm and PS at the highest value of 75 mm/s. At the mid of parametric value at exp number 5 (ID = 52%, PS = 61 mm/s and LT 0.22), as shown in Figure 6e, TS was found to be around 31.02 (see Figure 6h). Figure 6f shows the maximum and minimum TS values of the stress–strain curve. However, the PS remains less significant. Thus, the ID got dominated as an influencing factor on TS. The investigation coincides with the research performed by [46], where the PS was less significant than ID.

**Figure 7:**
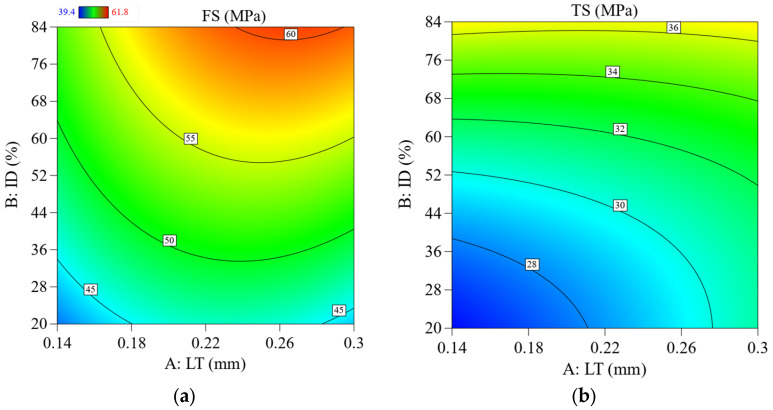
2D contour plots show the effect of the printing parameters and interaction on FS and TS; (**a**) effect of LT Vs. ID on FS, (**b**) effect of LT Vs. ID on TS.

Figure 7a shows the FS with contour graphs, showing that the LT and ID combinedly contributed to increasing the highest ID value and about 0.25 mm of the LT. However, the TS value did not significantly increase by changing the value of LT, as shown in Figure 7b.

### 3.3. Effect of Printing Parameters on Ra

**Figure 8:**
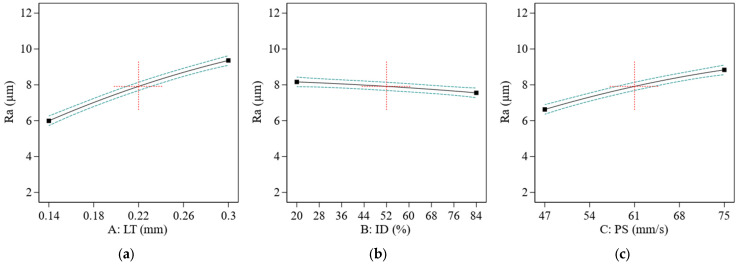
The effect of printing parameters on the Ra of the ABS polymer; (**a**) effect of LT on Ra, (**b**) effect of ID on Ra; (**c**) effect of PS on Ra. Red represents center point. Black is the average data line and green lines show the min and max of data.

When the LT and PS are both high, the surface of the printed item has a sharper appearance (see Figure 8a,b), and the layers emerge more, which results in increased Ra [63].

As seen in Figure 8a, an increase in LT caused the high staircase effect, resulting in Ra. In certain cases, the staircase effect may be minimized by adjusting the LT to a value that works the best value was significantly reduced by a decrease in LT. Its ID influenced the Ra of a 3D-printed item less. A smoother surface is shown in Figure 8b, as its D rises in the accompanying graph. This lower ID might result in rougher surface quality, as the extruded material has more opportunity to spread and produce rough or uneven layers, as seen in Figure 8b.

**Figure 9:**
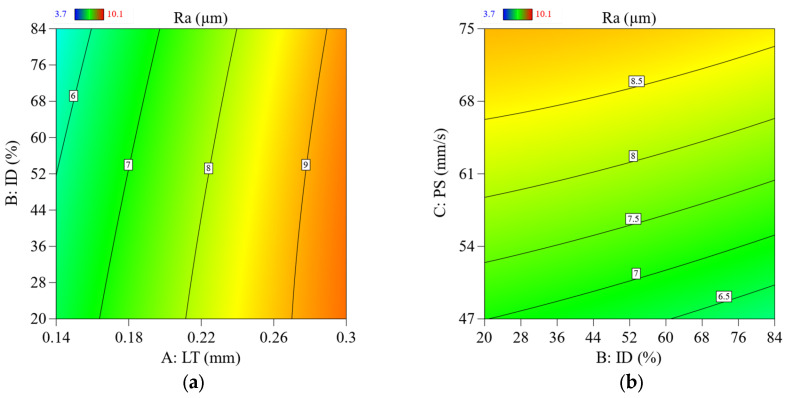
2D contour plots show the parameters’ effect and their interaction parameters on Ra; (**a**) effect of LT Vs. ID on Ra, (**b**) effect of LT Vs. PS on Ra.

**Figure 10:**
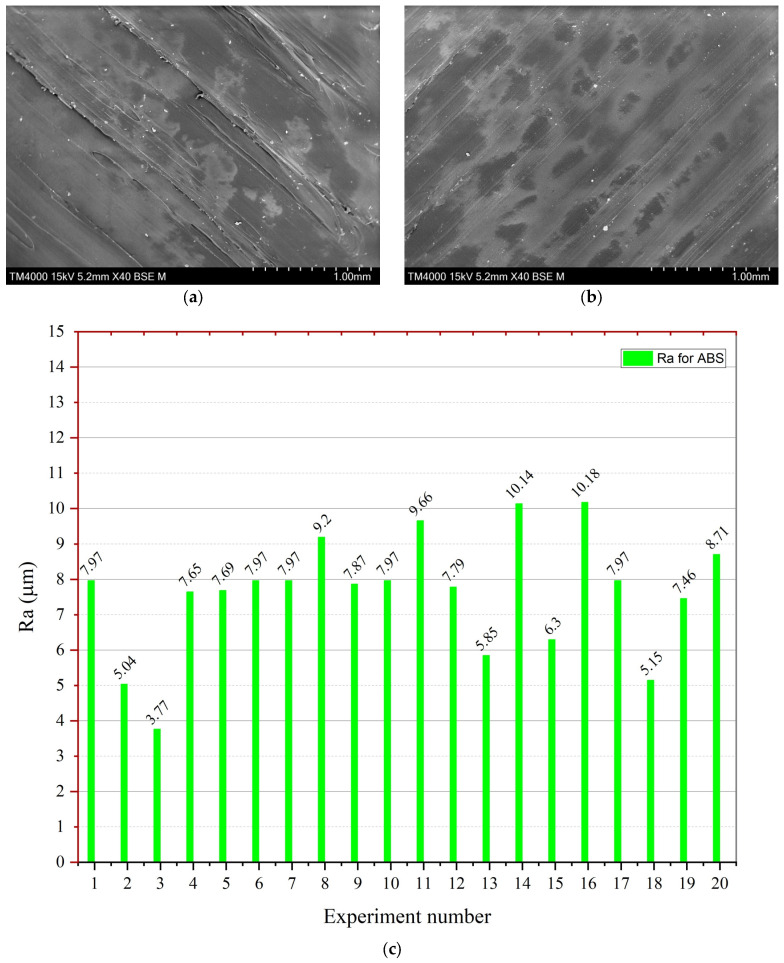
SEM analysis of Ra-ABS and experimental results; (**a**) SEM image of exp 16 (0.34 mm LT), (**b**) SEM image of exp 2 (LT = 0.14 mm, PS = 47 mm/s, ID 20%), and (**c**) graphical representation of each experimental performance parameter value of Ra.

Ra increased dramatically along with the increasing PS factor (Figure 8b). A high PS may create ringing artifacts and even shifting layers. The Ra value increased at lower values of ID. However, as shown in Figure 9a in contour graphs, it did not considerably influence the Ra, and the literature investigation results correspond with the literature findings [36]. After experimentation, the author found that the minimum Ra value was 3.77 µm, and the highest value for ABS was 10.18 µm (see Figure 10c). The contour graphs in Figure 9a,b show the lowest Ra value at the lowest LT, PS, and ID.

Figure 10a,b show the SEM images of the high Ra at experiment number 16—Figure 10a shows the results with a highly rough surface, while Figure 10b shows the lowest Ra of 3.77 µm.

### 3.4. Eco-Friendly 3D Printing

According to a report by Georgia State University’s School of Public Health and the Chemical Insights Research Institute, ABS polymer used in FFF 3DP can release fumes that emit a strong odor. These fumes can be harmful if inhaled. Decreasing print time and print energy can help minimize these fumes. This is because failed prints waste print time and material and limiting them will go a long way towards printing more efficiently. Additionally, heating plastic causes minor decomposition in the filament, producing particulate matter that can harm health. Therefore, reducing print time and energy can help minimize the amount of heated and decomposed plastic, thereby reducing the number of fumes and particulate matter released during printing.

**Figure 11:**
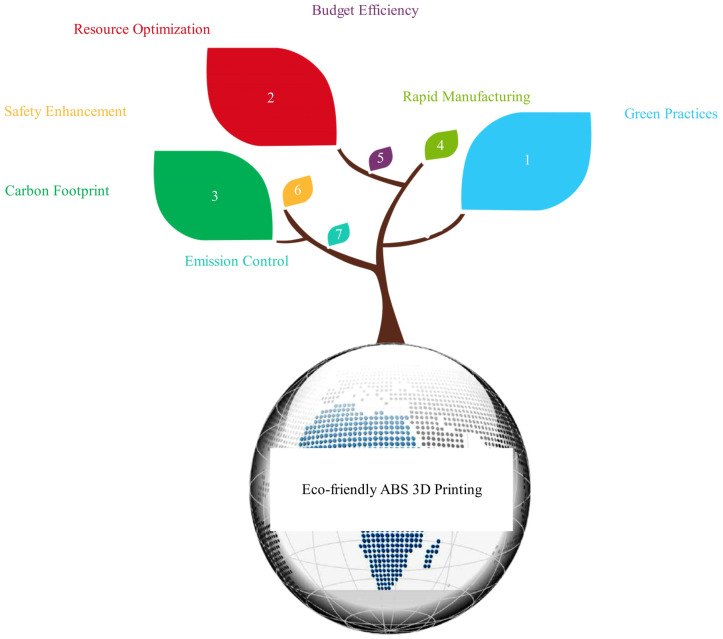
Eco-friendly ABS printing benefits.

Adopting an eco-conscious polishing strategy presents a forward-thinking approach to surface refinement, emphasizing environmentally considerate methods and materials (see Figure 11). By incorporating these green polishing techniques, manufacturers can lessen their ecological footprint, curtail waste production, and champion resource preservation. This mindset embraces using biodegradable, non-hazardous, or aqueous polishing compounds, in tandem with capitalizing on energy-conserving machinery and procedures. Furthermore, this green polishing philosophy may encompass integrating practices that curtail emissions, diminish energy demands, and confine the discharge of harmful substances. By wholeheartedly accepting eco-friendly polishing, industries can actively participate in fostering a more sustainable future and endorse conscientious manufacturing protocols while continuing to attain premium surface results on their merchandise.

**Figure 12:**
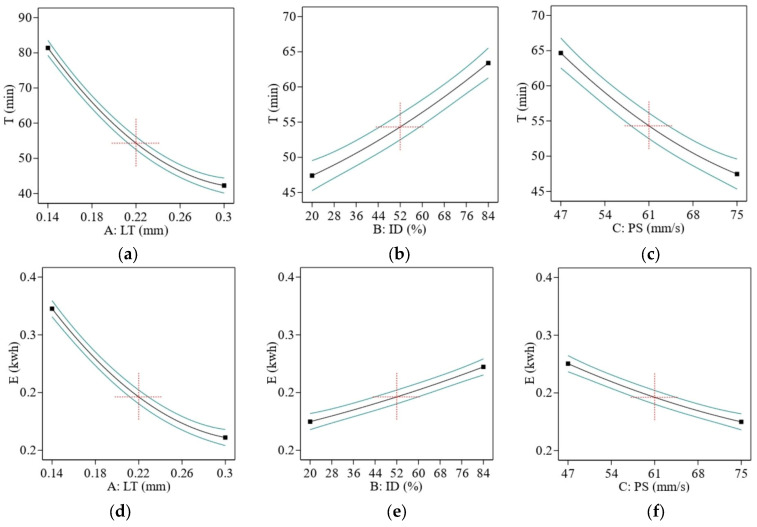
Effect of printing parameters on (**a**–**c**) for T and (**d**–**f**) for E. Red represents center point. Black is the average data line and green lines show the min and max of data.

Due to the staircase effect and the fact that the sample is completed in fewer cycles as LT increases, the printing time is reduced dramatically. Figure 12a,d shows that less E is consumed when controlling T.

How ID affects T in FFF printing is context-specific and subject to change based on several variables, such as the nature and size of the printed item, the kind of material being used, and the amount of precision and detail needed. If you increase the ID, the time constant will be longer; if you decrease it, the time constant will be shorter. Since FFF printing works by depositing layers of material on top of each other to build up the object, a larger ID requires more layers to be deposited to fill the object’s interior space. This additional material deposition takes time, resulting in a longer print time [64], as shown in Figure 12b,e.

In addition, the quality and durability of the final product may be modified by raising the ID. Stronger and longer-lasting items may be the outcome of higher ID. However, they might come at the expense of increased material requirements and more weight. This is especially relevant for mechanical components or working prototypes that will be put under stress or pressure.

**Figure 13:**
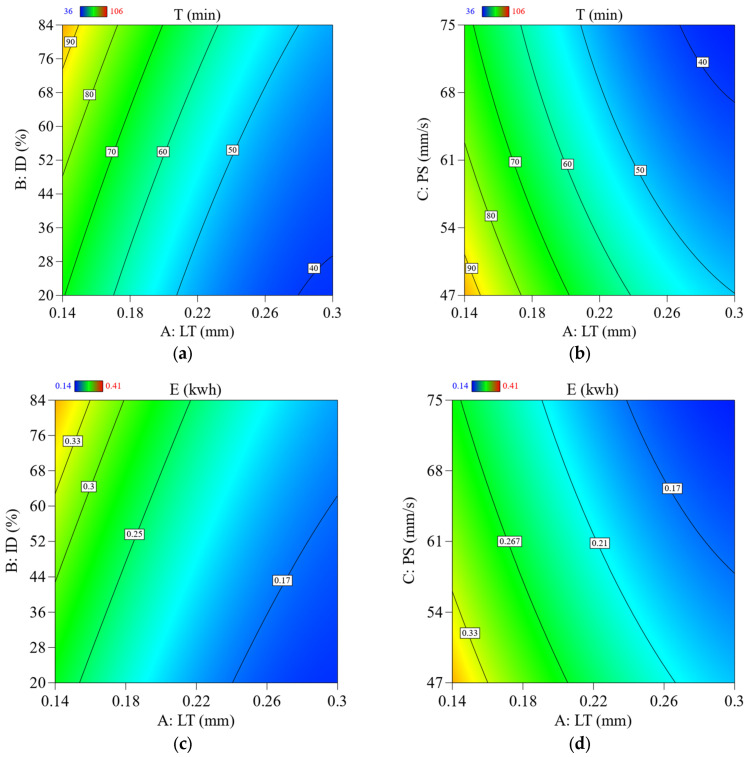
2D contour plots showing the effect of parameters and their interaction parameters on T and E; (**a**) effect of LT Vs. ID on T, (**b**) effect of LT Vs. PS on T, (**c**) effect of LT Vs. ID on E, (**d**) effect of LT Vs. PS on E.

The T decreased as the machine’s speed rose because the printer head could finish the cycle rapidly when working at high speed. The fact that the machine is only used briefly means that the E uses less energy, as shown in Figure 12c,f. The contour’s graphs in Figure 13 clearly show that for ABS, the lowest value of T and E was obtained using the highest value of LT, the lowest value of ID, and PS. The research agreed with previous research [48].

**Figure 14:**
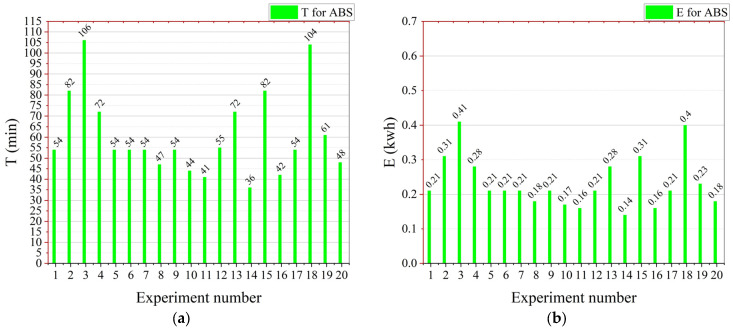
Graphical representation of the mechanical performance parameters values for every experiment: (**a**) for T-ABS and (**b**) for E-ABS.

Figure 14a,b shows the T and E for PLA, respectively. According to Figure 14a, experiment number 3 took the most T, due to the high ID = 84%, lowest LT = 0.14 mm, and lowest PS = 0.47. Due to the longer T, it also had higher E, which was around 0.41 kwh. The PLA sample took the least time at exp number 14 of 36 min due to the lowest ID, highest LT, and the highest level of the PS. Due to this reason, the E of experiment number 14 also had the least E of 0.14 kwh.

## 4. Multi-Optimization Using Response Surface Methodology

**Figure 15:**
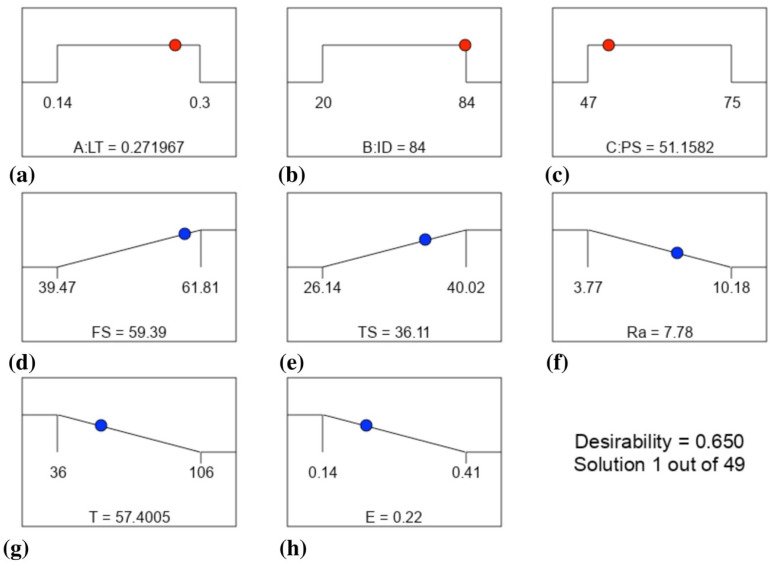
Optimization conditions and final performance parameters as the result of optimization: (**a**–**c**) for the parameters and (**d**–**h**) for the responses.

Optimal mechanical properties (TS, FS), smallest Ra, and shortest T for ABS polymer resulted from a multi-objective numerical optimization of process parameters. Numerical optimization may address multiple goals, with the resultant minimum and maximum values directly applicable to the parameter optimization. The red dots in Figure 15 represent the optimal levels of parameters, and the blue dots show the performance parameters. This optimization ramp was generated using the Design Expert software. The LT should be around 0.27 mm to obtain the optimal balance with ABS.

**Figure 16:**
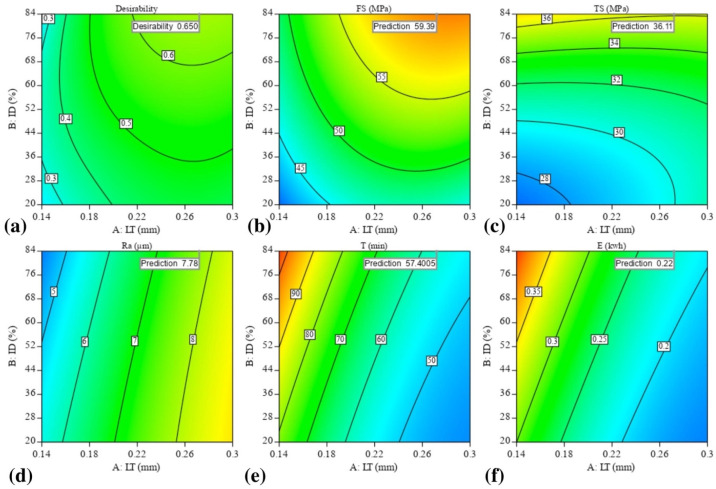
Numerical optimization plots show the region of optimal FFF parameters: (**a**) desirability, (**b**) FS optimization, (**c**) TS optimization, (**d**) Ra optimization, (**e**) T optimization, and (**f**) E optimization.

Maximum mechanical qualities, including FS of 59.39 MPa and TS of 36.11, lowest Ra of 7.78 m, lowest T of 57.4 min, and lowest E of 0.22 kwh, need ID and PS values of 84 percent and 51.1 mm/s, respectively. Figure 16 depicts contour graphs of the LT and ID relationships by highlighting the points when the PS and the permanent variable are at 51.1 mm/s values. Because the author set the LT to 0.27 (middle ground) and the ID to its maximum value at the top, the data points fell roughly in the end corner to the upper regions of the graph. It calculated a desirability of 0.65, which is quite good. We chose this optimization because it allowed us to get a balanced optimized sample with improved mechanical characteristics, a smoother surface, and a higher quality, even though we could have obtained the lowest values for Ra, T, and E at other places. This finding lends credence to the argument that the input parameters do have an impact on the outcomes. Finally, the enhanced mechanical properties (Ra, T, and E) created by the numerical optimization of the process parameters are tested for accuracy. This led to the development of three ABS specimens, which were used to double-check the experiment and determine the optimal parameters.

### Conformation Test

Laboratory trials with optimal parameter settings are compared to the projected values based on mathematical models, as indicated in the estimated proportion of errors given in Equation (8):

(8) $$\mathrm{Prediction}\;\mathrm{error}\;\mathrm{percentage}=\left|\frac{\mathrm{Actual}-\mathrm{predicted}}{\mathrm{actual}}\right|*100$$

It can be understood from Table 7 that the variation percentage between the predicted values and experimental values lies within 2.32%, 0.86%, 2.96%, 1.05%, and 4.55% for FS, TS, Ra, T, and E, respectively. Thus, the prediction performance of the model is satisfactory. This confirms an excellent accomplishment of results using the RSM-CCD.

## 5. Conclusions and Prospects

The following conclusions were drawn from the results of the experimentation:

- Inclusive multi-objective optimization of important performance parameters that are vital for industry (tensile strength (TS), flexural strength (FS), average surface roughness (Ra), print time (T), and energy consumption (E)) have been studied.
- Comprehensive investigations yielded contradictory optimized performance parameters, such as high FS and TS, low Ra value, and lowest T and E. The most crucial element in obtaining the desired Ra and T was LT (due to the staircase effect), whereas ID was the most crucial element in attaining the desired mechanical characteristics. PS also affected mechanical properties due to the polymer healing effects.
- Optimal printing settings combination for achieving FS, TS, Ra, T, and E for ABS were found at layer thickness LT = 0.27 mm, ID = 84%, and PS = 51.1 mm/s using the numerical multi-objective optimization. FS of 58.01 MPa, TS of 35.8 MPa, lowest Ra of 8.01 µm, lowest T of 58 min, and E of 0.21 kwh were attained using numerical multi-objective optimization. The variation percentage between the predicted and experimental values lies within 2.32%, 0.86%, 2.96%, 1.05%, and 4.55% for FS, TS, Ra, T, and E, respectively. Thus, the prediction implementation of the model is satisfactory.
- Reducing the T and E demonstrates that the FFF approach is feasible regarding power consumption, fuel efficiency, and controllable carbon emissions.
- The ABS mathematical models projected performance parameters findings and experimental results were all very close. When used for product quality testing, these data can be used as a guide to determine the best printing settings, saving time on trial and error.

## Figures and Tables

**Table 1:** Specifications of CR5-3D printer (credit: the3dstore.com, accessed on 10 April 2023).

Specification	Details
Layer Thickness	0.05–0.4 mm
Nozzle Diameter	Standard 0.4 mm (can be changed to 0.3/0.2 mm)
Filaments	1.75 mm PLA, ABS, PA6, TPU, Copper, Wood, Carbon Fiber
Print Speed	Normal: 60 mm/s, high: 100 mm/s
Printing Method	TF card/Online/Offline
Software Supporting	PROE, Solidworks, UG, 3D Max, Rhino 3D design
File Format	STL/OBJ/G-Code
Layers Software	Cura/Repetier-Host
Printing Size	300 × 225 × 320 mm
Print Temperature	Up to 270 °C
Power supply	230V
Bed Temperature	Up to 120 °C

**Table 2:** Comparison of standard ABS (credit: Kexcelled).

Properties	Values	Unit
Density	1.04	g/cm^3^
Flexural modulus	2000–3000	MPa
TS	30–40	MPa
Impact strength	Good	
Heat resistance	95–105 °C	°C

**Table 3:** Detailed parametric experimental design for the ABS polymer.

Exp #	LT (mm)	ID (%)	PS (mm/s)
1	0.22	52	61
2	0.14	20	47
3	0.14	84	47
4	0.22	100	61
5	0.22	52	61
6	0.22	52	61
7	0.22	52	61
8	0.22	52	82
9	0.22	52	61
10	0.22	4	61
11	0.34	52	61
*12*	*0.3*	*84*	*47*
13	0.22	52	40
14	0.3	20	75
15	0.14	84	75
16	0.3	84	75
17	0.22	52	61
18	0.1	52	61
*19*	*0.14*	*20*	*75*
20	0.3	20	47

**Table 4:** Performance parameters, mean, standard deviation, and ANOVA models were used for the investigation.

Performance Parameter	Name	Units	Observations	Min	Max	Mean	Std. Dev.	Ratio	Model
1	FS	MPa	20	39.47	61.81	50.07	6.75	1.57	Quadratic
2	TS	MPa	20	26.14	40.02	32.12	3.85	1.53	Quadratic
3	Ra	µm	20	3.77	10.18	7.62	1.69	2.70	Quadratic
4	T	min	20	36	106	60.80	19.69	2.94	Quadratic
5	E	kwh	20	0.14	0.41	0.2340	0.0754	2.93	Quadratic

**Table 5:** The standard deviation for each performance parameter at each point.

FS	TS	Ra	T	E
0.197525	1.5	0.017725	2.7	0.0105
2.0625	1.4105	0.252	4.1	0.0155
2.3	1.8995	0.1885	5.3	0.0205
3.0905	2.001	0.3825	3.6	0.014
2.736	1.554	0.3845	2.7	0.0105
2.695	1.552	0.3985	2.7	0.0105
2.68	1.4285	0.3985	2.7	0.0105
2.382	1.5435	0.46	2.35	0.009
2.6985	1.554	0.3935	2.7	0.0105
2.005	1.3995	0.3985	2.2	0.0085
2.5345	1.6995	0.483	2.05	0.008
2.899	1.781	0.3895	2.75	0.0105
2.573	1.565	0.2925	3.6	0.014
1.9735	1.558	0.507	1.8	0.007
2.776	1.8675	0.315	4.1	0.0155
2.8505	1.9055	0.509	2.1	0.008
2.7155	1.584	0.3985	2.7	0.0105
2.0665	1.4095	0.2575	5.2	0.02
2.0655	1.307	0.373	3.05	0.0115
2.265	1.527	0.4355	2.4	0.009

**Table 6:** Analysis results of the regression models.

Performance Parameter	R^2^	Adj-R^2^	Pre-R^2^	Precision	F-Value	Lack-of-Fit	Model *p*-Value
FS	97.68	95.60	81.97	23.09	46.09	0.004	<0.0001
TS	95.82	92.05	82.45	19.59	25.45	0.556	<0.0001
Ra	98.78	97.67	90.86	34.22	89.64	0.01	<0.0001
T	99.43	98.93	95.82	71.63	196.35		<0.0001
E	99.27	98.61	94.64	44.33	150.26		<0.0001

**Table 7:** Predicted process parameters, performance parameters, experimental performance parameters, and error by RSM optimization.

Predicted Process Parameters			Predicted Performance Parameters			Experimental Performance Parameters			Error %
Name	Unit	Value	Name	Unit	Value	Name	Unit	Value	Value
LT	mm	0.27	FS	MPa	59.39	FS	MPa	58.01	2.32
ID	%	84	TS	MPa	36.11	TS	MPa	35.8	0.86
PS	mm/s	51.1	Ra	μm	7.78	Ra	μm	8.01	2.96
			T	min	57.4	T	min	58	1.05
			E	kwh	0.22	E	kwh	0.21	4.55

## Data Availability

Not applicable.
